# Supplementary material for: Patterns of Intron Gain and Loss in Fungi
Source: PLoS Biol. 2004 Nov 30;2(12):e422. doi: 10.1371/journal.pbio.0020422 (PMC532390; doi:10.1371/journal.pbio.0020422)
Supplement: Table S1 — Also available at http://genes.mit.edu/NielsenEtAl/. (4.3 MB ZIP). [file pbio.0020422.st001.zip › NielsenEtAl/html/1286.html]

AN4163.1.NCU05810.1.MG04719.1.FG09870.1


```
 CLUSTAL W (1.82) Multiple Sequence Alignments - Introns Inserted


Sequence 1: MG04719.1	316 aa
Sequence 2: FG09870.1	316 aa
Sequence 3: NCU05810.1	316 aa
Sequence 4: AN4163.1	316 aa
Alignment Length: 316 aa
Number Identitical Residues: 281 aa
Alignment Score (without introns) 11351


MG04719.1 	MAEQLILKGTLEGH0NGWVTSLATSMEN2PNMLLSSSRDKTLIIWNLTRDETSYGYPKRS
NCU05810.1	MAEQLILKGTLEGH0NGWVTSLATSLEN2PNMLLSGSRDKSLIIWNLTRDETSYGYPKRR
FG09870.1 	MAEQLILKGTLEGH0NGWVTSLATSMEN2PNMLLSASRDKTLIIWNLTRDETQYGYPKRS
AN4163.1  	MAEQLVLRGTLEGH~NGWVTSLATSLEN2PNMLLSGSRDKTLIIWNLTRDEQAYGYPKRS
          	*****:*:****** **********:** ******.****:**********  ****** 

MG04719.1 	LKGHSHIVSDC0VISSDGAYALSASWDKTLRLWELATGTTTRRFVGHTNDVLSVSFSADN
NCU05810.1	LHGHSHIVSDC0VISSDGAYALSASWDKTLRLWELSTGTTTRRFVGHTNDVLSVSFSADN
FG09870.1 	LHGHSHIVSDC0VISSDGAYALSASWDKTLRLWELATGTTTRRFVGHTNDVLSVSFSADN
AN4163.1  	LEGHSHIVSDC0VISSDGAYALSASWDKSLRLWELSSGQTTRTFVGHTNDVLSVSFSADN
          	*.********* ****************:******::* *** *****************

MG04719.1 	RQIVSGSRDRSIKLWNTLGDCKYTITEKGHSEWVSCVRFSPNPQNPVIVSSGWDKLVK0V
NCU05810.1	RQIVSGSRDRSIKLWNTLGDCKFTITEKGHTEWVSCVRFSPNPQNPVIVSSGWDKLVK0V
FG09870.1 	RQIVSGSRDRTIKLWNTLGDCKYTITDKGHTEWASCVRFSPNPQNPVIVSAGWDKLVK0V
AN4163.1  	RQIVSGSRDRTIKLWNTLGDCKYTITDKGHTEWVSCVRFSPNPQNPVIVSAGWDKLVK0V
          	**********:***********:***:***:**.****************:******* *

MG04719.1 	WELSSCKLQTDHIGHTGYINTVTISPDGSLCASGGKDGTTMLWDLNESKHLYSLNANDEI
NCU05810.1	WELSSCKLQTDHIGHTGYINAVTISPDGSLCASGGKDGTTMLWDLNESKHLYSLNANDEI
FG09870.1 	WELSTCKLQTDHIGHTGYINTVTISPDGSLCASGGKDGTTMLWDLNESKHLYSLNANDEI
AN4163.1  	WELASCRLQTDHIGHTGYINTVTISPDGSLCASGGKDGVTMLWDLNESKHLYSLHAGDEI
          	***::*:*************:*****************.***************:*.***

MG04719.1 	HALVFSPNRYWLCAATASSIIIFDLEKKSKVDELKPEFAAVGKKSREPECISLAWSADGQ
NCU05810.1	HALVFSPNRYWLCAATSSSIIIFDLEKKSKVDELKPEFQNIGKKSREPECVSLAWSADGQ
FG09870.1 	HALVFSPNRYWLCAATASSIIIFDLEKKSKVDELKPEFPAAGKKSREPECVSLAWSADGQ
AN4163.1  	HALVFSPNRYWLCAATSSSITIFDLEKKSKVDELKPEYIEKGKKSREPECVSLAWSADGQ
          	****************:*** ****************:   *********:*********

MG04719.1 	TLFAGYTDNIIRAWGVMSRA
NCU05810.1	TLFAGYTDNIIRAWGVMSRA
FG09870.1 	TLFAGYTDNIIRAWGVMSRA
AN4163.1  	TLFAGYTDNKIRAWGVMSRA
          	********* **********
```
